# Silicon Dioxide Nanoparticles-Based Amelioration of Cd Toxicity by Regulating Antioxidant Activity and Photosynthetic Parameters in a Line Developed from Wild Rice

**DOI:** 10.3390/plants13121715

**Published:** 2024-06-20

**Authors:** Humera Ashraf, Fozia Ghouri, Jiabin Liang, Weiwei Xia, Zhiming Zheng, Muhammad Qasim Shahid, Xuelin Fu

**Affiliations:** 1 State Key Laboratory for Conservation and Utilization of Subtropical Agro-Bioresources, South China Agricultural University, Guangzhou 510642, China; humeraa49@gmail.com (H.A.); foziaghouri@scau.edu.cn (F.G.); 1634768793@stu.scau.edu.cn (J.L.); 13087379566@stu.scau.edu.cn (W.X.); zhengzhiming2022@stu.scau.edu.cn (Z.Z.); 2 Guangdong Provincial Key Laboratory of Plant Molecular Breeding, College of Agriculture, South China Agricultural University, Guangzhou 510642, China

**Keywords:** *OsABC*, *OsHMA3*, ROS, cadmium, gene expression

## Abstract

An extremely hazardous heavy metal called cadmium (Cd) is frequently released into the soil, causing a considerable reduction in plant productivity and safety. In an effort to reduce the toxicity of Cd, silicon dioxide nanoparticles were chosen because of their capability to react with metallic substances and decrease their adsorption. This study examines the processes that underlie the stress caused by Cd and how SiO_2_NPs may be able to lessen it through modifying antioxidant defense, oxidative stress, and photosynthesis. A 100 μM concentration of Cd stress was applied to the hydroponically grown wild rice line, and 50 μM of silicon dioxide nanoparticles (SiO_2_NPs) was given. The study depicted that when 50 μM SiO_2_NPs was applied, there was a significant decrease in Cd uptake in both roots and shoots by 30.2% and 15.8% under 100 μM Cd stress, respectively. The results illustrated that Cd had a detrimental effect on carotenoid and chlorophyll levels and other growth-related traits. Additionally, it increased the levels of ROS in plants, which reduced the antioxidant capability by 18.8% (SOD), 39.2% (POD), 32.6% (CAT), and 25.01% (GR) in wild rice. Nevertheless, the addition of silicon dioxide nanoparticles reduced oxidative damage and the overall amount of Cd uptake, which lessened the toxicity caused by Cd. Reduced formation of reactive oxygen species (ROS), including MDA and H_2_O_2_, and an increased defense system of antioxidants in the plants provided evidence for this. Moreover, SiO_2_NPs enhanced the Cd resistance, upregulated the genes related to antioxidants and silicon, and reduced metal transporters’ expression levels.

## 1. Introduction

Heavy metals (HMs) are found in the environment, are considered as harmful substances, and may become bio-concentrated by animals and plants and ingested into the body through the food web [1,2], having a negative impact on health [3]. Nevertheless, as industry and urbanization grow more quickly, water and land bodies become polluted with a wide range of HMs [4]. Moreover, Cadmium (Cd) is found in polluted soils and is extremely cytotoxic, having half-life of up to 30 years. As per China’s soil heavy metals pollution status, Cd appears to be one of the most common contaminants, with soil Cd contamination exceeding the standard rate of 7.0% [5]. Previous research has showed that plants exposed to Cd toxicity have shorter rhizomes, lower biomass, less photosynthesis, and higher ROS production [6]. Therefore, developing eco-friendly techniques to stop Cd buildup and lessen crop Cd detrimental effects is essential [7]. While many strategies are being used worldwide to mitigate and remediate heavy metal stress, some gaps still need to be filled, including those related to ecological suitability, economic benefits, and environmental sustainability.

Nanotechnology has drawn consideration due to its numerous uses in different fields of research, such as medicine, energy, and materials; because of its unique qualities, such as its small size, ease of use, and ability to be applied in low concentrations, nanotechnology holds great promise for advancing both biotechnology and agriculture [8,9]. Nanotechnology has emerged as a viable solution that can address the rising need to tackle HMs pollution and have a positive effect on plant growth [10,11]. Many applications for nanomaterials exist, including herbicides, insecticides, fertilizers, growth-stimulating agents for plants, and agents that decrease HMs stress in crops [9,12,13,14,15,16,17]. Nanoparticles have a crucial role in environment protection, soil fertility, and sustainable agricultural growth as they may minimize the loss of nutrients, reduce pathogens, facilitate the growing of seeds, boost chlorophyll contents in plants, raise crop yields, and strengthen plant resilience to toxic conditions [18,19]. Additionally, nanomaterials have great potential for environmental safety and mine soil recovery [15]. Silicon dioxide nanoparticles (SiO_2_NPs) have emerged as viable agents that can fulfill the increasing requirement for crop nutrition, limit plant Cd accumulation and uptake, and prevent Cd toxicity [20,21]. Silicon dioxide nanoparticles have been analyzed as a promising type of nanomaterial that has the potential to address many stressors, including heavy metal toxicity in polluted soil [10,22,23,24]. Furthermore, compared to conventional silicon fertilizers, SiO_2_NPs can more effectively reduce Cd stress [10]. The use of silicon dioxide nanoparticles in Cd toxicity, plant growth, photosynthesis parameters, and chlorophyll contents can be enhanced, and the concentration of Cd within plants can be reduced [20,21,23,25]. Following the application of 5–25 mM (mmol/L) silicon dioxide nanoparticles during the anthesis stage, the amount of Cd adsorption in rice plants decreased significantly [10].

Rice is the source of feeding to approximately 50 percent of the global population, making it one of the most significant food globally [26]. Different approaches were used to increase the yield, resistance, and genetic diversity of cultivated rice, such as hybrid rice, through the development of chromosomal segment substitution lines by crossing cultivated rice with wild rice [27,28,29]. Chromosome-segment substitution lines (CSSLs), make it possible to analyze target quantitative trait loci (QTLs) with greater accuracy. The genotypes that makeup CSSLs populations are produced by recurrent backcrossing and marker-assisted selection (MAS) and contain a small number of chromosomal segments from a donor that are replaced by the genetic background of a recipient. CSSLs containing a single donor substitution segment are referred to as near-isogenic lines or single segment substitution lines (SSSLs) [28]. Moreover, single segment substitution lines of wild rice act as a bridge between genetic diversity of wild rice and the vital agronomic traits of cultivated rice [27,28]. By using this genetic diversity, rice varieties can be enhanced and made more resilient to the demands of modern agriculture by promoting global food security. Furthermore, across Central and South America, *Oryza glumaepatula* is a member of wild rice with an AA genome and is very important in rice production [30].

The current study was conducted to evaluate wild rice’s ability to withstand impairments caused by Cd, as well as its ability to withstand the counteracting effects of SiO_2_NPs. We assessed the effects of SiO_2_ NPs and Cd doses on the amount of MDA, H_2_O_2_ accumulation, electrolyte leakage, photosynthesis activity, antioxidant enzyme parameters, and chlorophyll contents. To assess the degree of harm and the protection offered by SiO_2_NPs over Cd toxicity, the growth characteristics of wild rice and the level of Cd were also noted. This study shows how SiO_2_NPs can help rice to develop better under HMs toxicity, especially when it comes to tolerating the stress of Cd.

## 2. Results

### 2.1. Characterization of SiO_2_NPs

**Figure 1:**
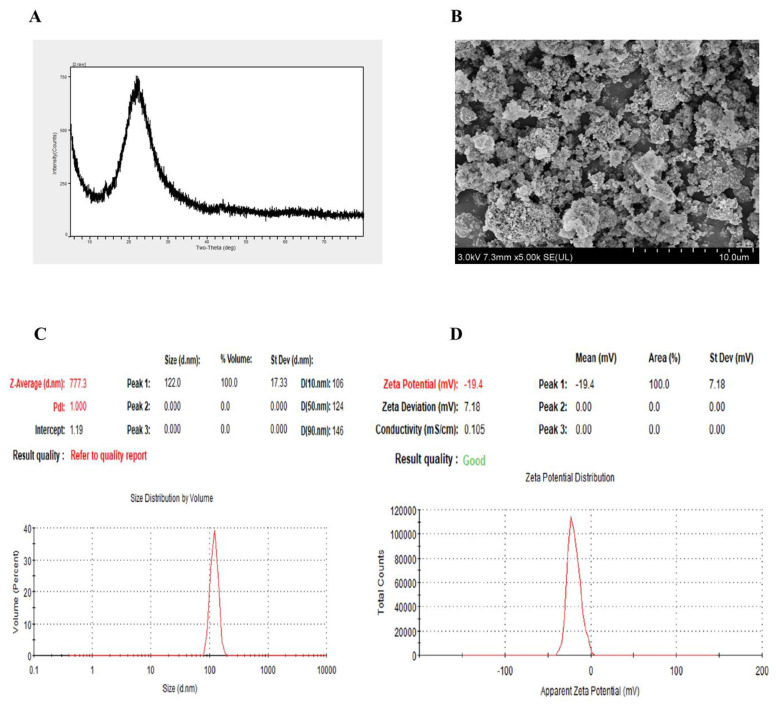
Characterization of silicon dioxide nanoparticles (SiO_2_NPs). (**A**) X-ray diffraction of SiO_2_NPs. (**B**) SiO_2_NPs observed under the scanning electron microscope. (**C**) DLS (Dynamic Light Scattering) of SiO_2_NPs. (**D**) Zeta potential of SiO_2_NPs.

In this study, we utilized commercially available silicon dioxide nanoparticles (SiO_2_NPs) with a purity of 99.9%. The X-ray diffraction analysis showed that the silicon dioxide nanoparticles were free from impurities and exhibited an amorphous structure, including silicon dioxide (SiO_2_) (Figure 1A). Furthermore, no extra peaks were detected. The size distribution analysis revealed that SiO_2_NPs were aggregated, with evidence of significant particle clustering (Figure 1B). The average particle size of silicon dioxide nanoparticles was 777.3 d.nm, and the polydispersity index (PDI) was 1.000 (Figure 1C). The Zeta potential of silica nanoparticles was −19.4 ± 7.18 mV (Figure 1D).

### 2.2. Treatment of SiO_2_NPs Enhanced Plant Growth under Cd Toxicity

**Figure 2:**
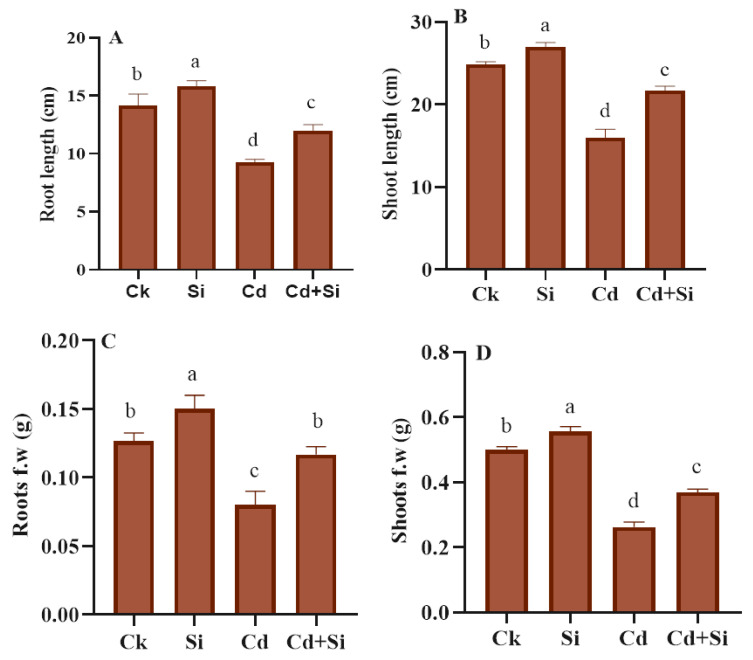
SiO_2_NPs effect on growth attributes (**A**–**D**) of wild rice under Cd toxicity. Error bars indicate the standard error and the significance level is depicted by using different letters with LSD statistical test (*p* ≤ 0.05). FW, fresh weight, Ck: control, Si: SiO_2_NPs, Cd: Cadmium, Cd + Si: Cadmium + SiO_2_NPs.

The findings of different growth-related parameters of the plants, such as shoot length (SL), root length (RL), fresh weight of shoots (FWS), and fresh weight of roots (FWR) are shown in Figure 2. Under Cd stress, wild rice root length and shoot length, FWS (fresh weight of shoots), and FWR (fresh weight of roots) declined by 35%, 36%, 39%, and 47%, respectively, as compared to the control plants. Nevertheless, the use of SiO_2_NPs under Cd stress in wild rice seedlings significantly increased the above mentioned all growth parameters as compared to Cd stress alone treated plants. The treatment of SiO_2_NPs considerably enhanced the RL 68.88%, SL 80.2%, FWS 71.17%, and FWR 72.72% in Cd toxic plants.

### 2.3. Effect of SiO_2_NPs on Photosynthetic Characteristics of Wild Rice under Cd Stress

**Figure 3:**
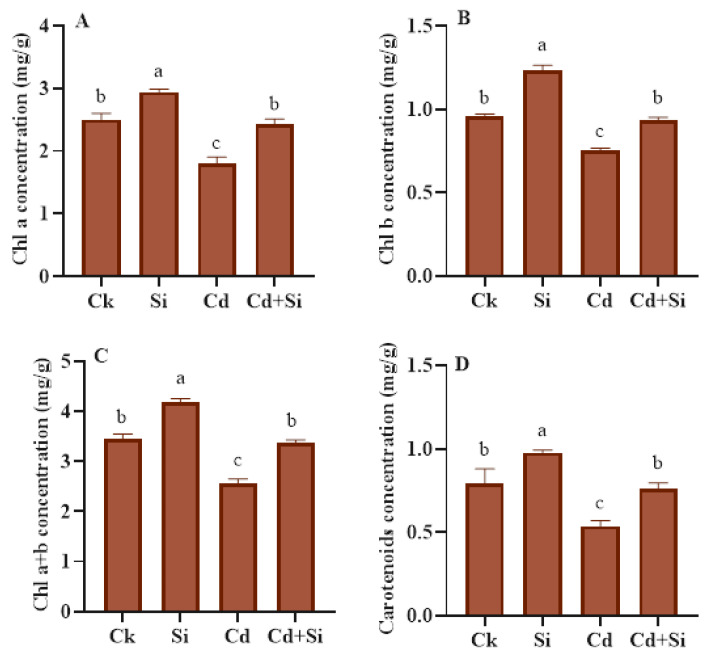
SiO_2_NPs effects on carotenoids and chlorophyll concentration of wild rice under Cd toxicity (**A**–**D**). Error bars indicate the standard error and the level of significance is depicted by using different alphabetical letters by LSD statistical test (*p* ≤ 0.05).

The findings showed the detrimental effect of Cd stress on photosynthetic parameters in wild rice and its significant amelioration by using SiO_2_NPs (Figure 3). Chlorophyll a, chlorophyll b, carotenoids, and chlorophyll a + b contents decreased by 28%, 22%, 26%, and 32%, respectively, under Cd stress compared to control plants. However, SiO_2_NPs + Cd treatment enhanced the carotenoids by 41.61%, chlorophyll a by 35.18%, chlorophyll a + b by 32.15%, and chlorophyll b contents by 24.88% compared to Cd toxic plants.

### 2.4. Effect of SiO_2_NPs on MDA and H_2_O_2_ Levels in Wild Rice Subjected to Cd Stress

**Figure 4:**
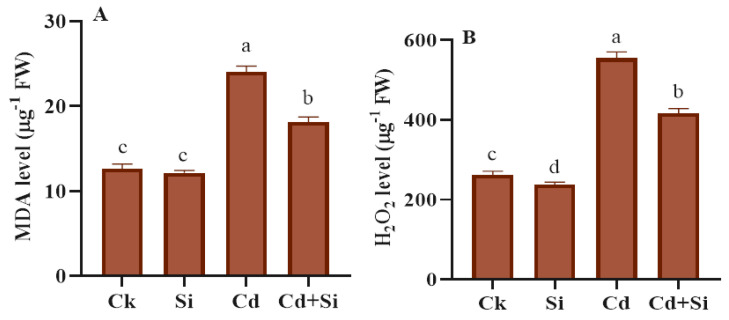
SiO_2_NPs effect on MDA and H_2_O_2_ level in wild rice under Cd toxicity. (**A**) MDA level. (**B**) H_2_O_2_ level. Error bars indicate the standard error, and the level of significance is depicted by using different letters using LSD statistical test (*p* ≤ 0.05).

The reactive oxygen species stress was induced through Cd stress to explore the effect of SiO_2_NPs in decreasing Cd oxidative stress in wild rice (Figure 4). The analyses showed that Cd stress promotes the H_2_O_2_ and MDA levels, notably in Cd-stressed rice plants. The MDA level was promoted by 91% in the Cd stressed group as compared to the control group. Likewise, the H_2_O_2_ concentration was enhanced by 111% in Cd treated plants compared to control plants. In addition, SiO_2_NPs application reduced the H_2_O_2_ and the MDA by 25% and 24%, respectively, as compared to Cd toxic plants alone.

### 2.5. Effects of Antioxidant Enzymatic Defense in Wild Rice under Cadmium Toxicity

**Figure 5:**
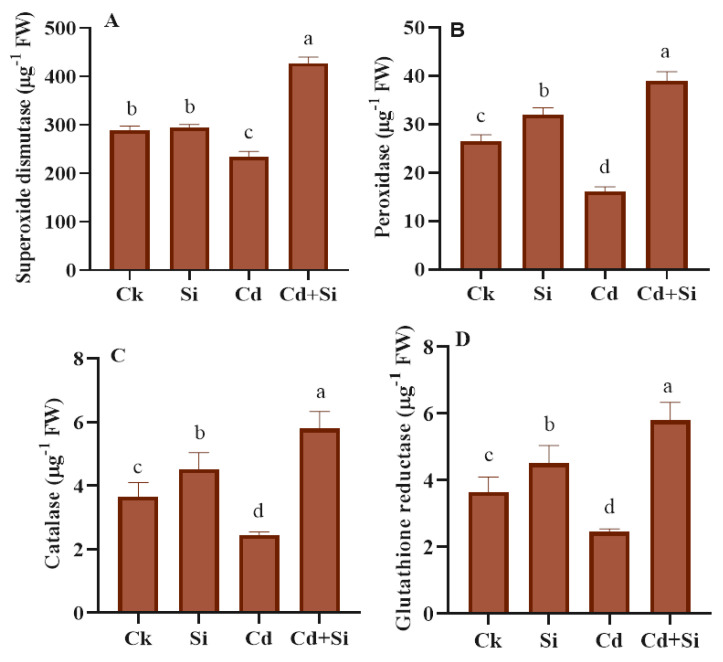
Effect of SiO_2_NPs on antioxidant defense activity in wild rice under Cd toxicity. (**A**) Superoxide dismutase. (**B**) Peroxidase. (**C**) Catalase. (**D**) Glutathione reductase. Error bars indicate the standard error, and the level of significance is depicted by using different alphabetical letters by the use of the LSD statistical test (*p* ≤ 0.05).

In wild rice, the antioxidant defense system, including CAT, POD, SOD, and GR functions, declined due to Cd toxicity in rice plants. However, the application of SiO_2_NPs + Cd significantly increased their activities by 82.24%, 141.61%, 136.73%, and 129.54% compared to plants treated just with Cd (Figure 5). POD, SOD, CAT, and GR activity reduced significantly by 25%, 34%, 22%, and 49.28% under Cd exposure in contrast to untreated plant samples and was promoted by the use of SiO_2_NPs.

### 2.6. Effects of SiO_2_NPs on Cd and Si Concentration in Wild Rice

**Figure 6:**
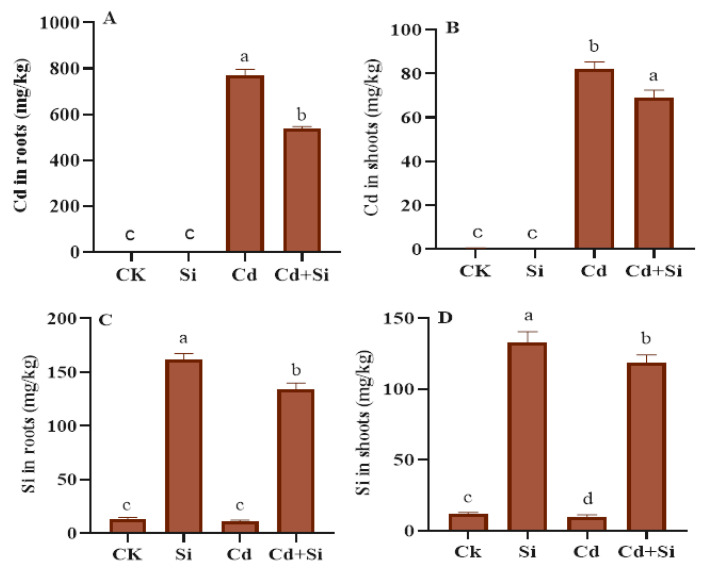
Effect of SiO_2_NPs on Cd and Si contents in wild rice under Cd toxicity. (**A**) Cd in roots. (**B**) Cd in shoots. (**C**) Si in roots. (**D**) Si in shoots. Error bars indicate the standard error, and the level of significance is depicted by different alphabetical letters using the LSD statistical test (*p* ≤ 0.05).

The wild rice plants treated with SiO_2_NPs showed a lower level of Cd and a higher level of Si uptake in roots and shoots under Cd toxicity (Figure 6). The results depicted that the application of SiO_2_NPs decreased the Cd contents significantly to 538.11 mg/kg in roots and 69.09 mg/kg in shoots compared to plants with Cd toxicity (roots with 771.71 mg/kg and shoots with 82 mg/kg). The Si uptake in shoots declined by 9.95 mg/kg, and in roots was 10.95 mg/kg as compared to control plants (11.54 mg/kg in shoots and 12.90 mg/kg in roots). Nevertheless, in SiO_2_NP + Cd treated plants, Si was enhanced to 132.86 mg/kg (shoots) and 162.05 mg/kg (roots) as compared to Cd alone plants (9.95 mg/kg (shoots, 10.96 mg/kg (roots). However, the concentration of Cd was still high in SiO_2_ + Cd wild rice compared to the control group (Figure 6).

### 2.7. Expression Level of Transporters and Genes

**Figure 7:**
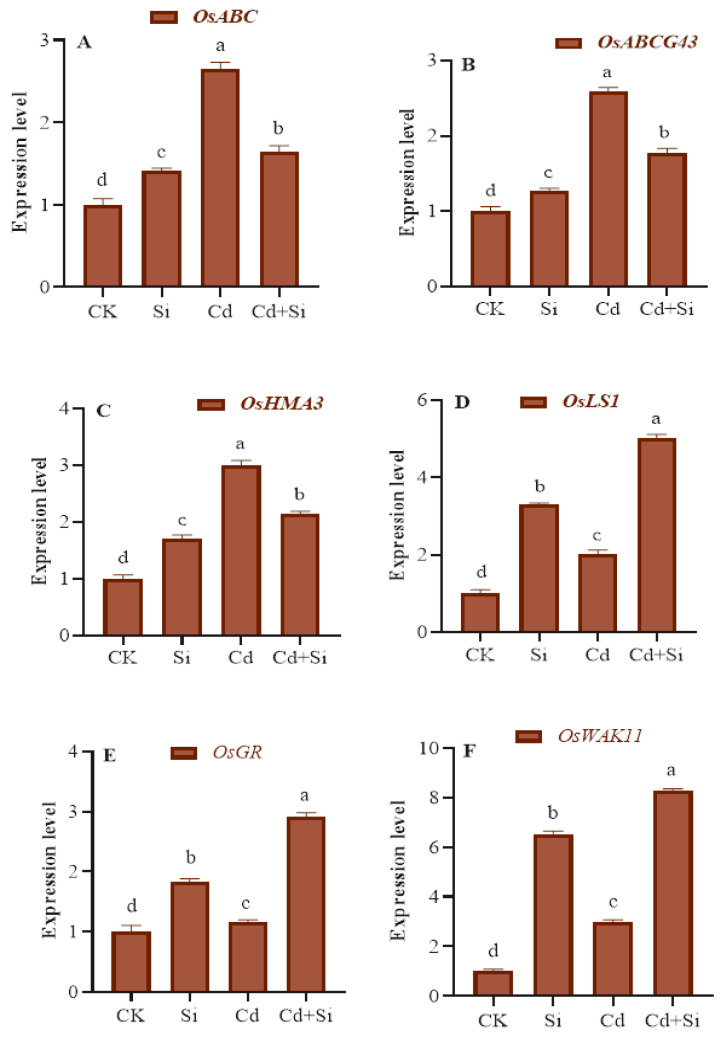
Effect of SiO_2_NPs and Cd on the expression levels of metal transporters and other genes in wild rice under Cd toxicity (**A**–**F**). Error bars indicate the standard error, and the level of significance is depicted by different alphabetical letters using the LSD statistical test (*p* ≤ 0.05).

The supplementation of Si and Cd caused a significant change in their transporters and gene expression levels (Figure 7). The expression levels of *OsABC*, *OsABCG43*, and *OsHMA3* were the highest under Cd toxicity but decreased notably with SiO_2_NPs (Figure 7). The expression effects of *OsGR*, *OsLS1*, and *OsWAK11* were significantly enhanced after the use of SiO_2_NPs.

## 3. Discussion

The development of innovative strategies for agrochemical products by the use of nanoparticles to increase their effectiveness and absorption in certain crop zones has continued [31,32,33]. Through the application of nanotechnology, precision agriculture is experiencing a revolution that will enable more economical, sustainable, and efficient use of resources [34]. Moreover, SiO_2_NPs can interact with plants directly or indirectly, which can result in improved crop quality, increased enzymatic activity, a stronger and more effective shoot system, and increased photosynthetic efficiency [32]. On the other hand, one of the biggest obstacles to crop productivity is metallic stress, especially cadmium (Cd) stress. This poisonous and undesirable heavy metal is quickly taken by plants, leading to the manifestation of symptoms including chlorosis, browning of the roots, leaf blight, and stunted development, which greatly decline agricultural production [35]. A variety of approaches, including the use of NPs (Nanoparticles), have been devised to mitigate the harmful impacts of cadmium [12]. It has been shown that silicon (Si) reduces plant toxicity symptoms triggered by HMs, including Cd [36]. Furthermore, compared to traditional silicon-based fertilizers, using SiO_2_NPs is a more effective way to reduce Cd stress [10]. This paper demonstrated the valuable impacts of SiO_2_NPs under Cd stress in rice plants of *Oryza glumaepatula*. Furthermore, SiO_2_NPs facilitated the bio-chemical and physiological defense mechanisms of plants. The results of our study revealed that the application of silicon dioxide nanoparticles diminished the Cd triggered phytotoxicity in wild rice. This was demonstrated by better morphological traits of rice plants under Cd+ SiO_2_NPs treatment than Cd treated plants only (Figure 2A–D). Our results were consistent with those of previous research [1,37], which proved the same findings but in different plants. By interfering with the physiological and molecular processes of plants, Cd may prevent plants from growing [38,39]. Several researchers have found that supplementation of NPs to crops under heavy metal stress increased crop development, using a variety of methods including the boosting of mineral nutrition or the reducing of metallic stress in agriculture [40,41,42,43,44].

Evaluating photosynthetic pigments using an efficient and quick method may be helpful in determining how effectively plants are developing, especially in harsh environments [45]. According to the current research, silicon dioxide nanoparticles increased the amount of chlorophyll and carotenoid levels in the wild rice line (Figure 3A–D). In previous research, when crops were subjected to Cd toxicity, seedlings of barley and wheat showed increased photosynthetic activity after using silicon dioxide nanoparticles (SiO_2_NPs) [1,12]. The plants with nanoparticle treatment showed improved photosynthesis rates compared to those without nanoparticle treatment [46].

One of the notable ways that Cd is hazardous to rice plants is the damage of lipids and proteins; additionally, it even ruptures the membranes of the plants [47]. An overabundance of reactive oxygen species causes oxidative disruptions within cells, inhibiting growth and causing damage [48]. The Cd oxidative explosion in crops may be linked to increased MDA and EL readings and decreased antioxidant levels in the control group [49]. The current study depicted that SiO_2_NPs decreased H_2_O_2_ (hydrogen peroxide) and MDA (malondialdehyde) levels in plants of *Oryza glumaepatula*. Consequently, the control tissues clearly showed that, with the reduced biomass and development of crops under Cd effect, the administration of SiO_2_NPs greatly reduced these adverse effects of Cd, which triggered the production of ROS in wild rice plants (Figure 4A,B).

Plants have evolved complex defensive systems, which use enzymes like peroxidase (POD), superoxide dismutase (SOD), ascorbate peroxidase (APX), and catalase (CAT) to remove unwanted ROS within their cells. Superoxide dismutase contributes to the dismutation of O^2−^ to produce hydrogen peroxide, whereas CAT can degrade H_2_O_2_ into H_2_O and O_2_ through a two-step process. Furthermore, POD aids in the decomposition of lipid peroxides, while APX uses the ascorbate–glutathione cycle to detoxify H_2_O_2_ against redox stress [50]. To verify that oxidative damage was being generated, the antioxidant enzyme activity was assessed. Moreover, the same findings have been identified in previous research [1], which reported that the enhanced ROS contents and the widespread production of the H_2_O_2_ scavenging mechanism through antioxidant enzymes happened due to heavy metal stress. In the current research, the use of silicon dioxide nanoparticles could mitigate the oxidative damage caused by Cd toxicity, as it promoted the activity of several antioxidant enzymes in wild rice under Cd toxicity (Figure 5A–D). In accordance with the findings reported in previous research, the high concentration of antioxidants in plants could play various roles, like detoxifying harmful ROS, preserving the integrity of the membrane, stabilizing the enzyme activities, and controlling the osmotic equilibrium. All of these enhance the plant’s resistance to the harmful effects of Cd [51]. Prior research has shown that the addition of SiO_2_NPs improved the capability of antioxidant enzymes in different plants, such as in wheat seedlings [52], wheat grains at various plant development stages under Cd toxicity [25,35], barley seedlings under Cd toxicity [1], bamboo plant under lead toxicity [53], hybrid rice under water regime surroundings [54], and rice under Cd and Pb toxicity [55,56]. Our findings suggested that the supply of SiO_2_NPs might lower oxidative damage by declining the Cd toxicity and enhancing the antioxidant capacity in wild rice. 

Further published studies have shown that silicon dioxide nanoparticles could reduce the amounts of Cd in several plants [20,57,58,59]. The use of SiO_2_NPs also declines the metal concentration of rice seedlings and lowers the translocation of metal in shoots from roots [10]. The use of SiO_2_NPs has been shown to reduce the uptake of Cd and increase the uptake of Si in plants [23,55]. Our findings show that the Cd contents significantly decreased in wild rice under Cd toxicity after applying SiO_2_NPs. However, the Si contents were enhanced considerably in these plants in the same condition and with the same treatment (Figure 6A–D). This hydroponic application of SiO_2_NPs decreased the Cd translocation to shoots; additionally, it may be linked to an augmented plant biomass in Cd stress. This might have the effect of dilution, increasing biomass at the same concentration. To fully understand this mechanism, more in-depth research will be needed in the future.

Many genes that are important for the movement and buildup of heavy metals within rice are coordinated in their activity throughout the process of HMs [55]. According to our findings, the application of SiO_2_NPs successfully suppressed the absorption of Cd by declining the expression of *OsABC*, *OsABCG43,* and *OsHMA3* as well as enhancing the expression level of *OsGR* and *OsWAK11* as compared to Cd treated plants alone (Figure 7). Consistent with the findings from prior studies, the upregulated expression of metal transporters and nanoparticles in Cd exposure implies their possible involvement in Cd absorption and transport [55,60]. Following the use of SiO_2_NPs in our study, we observed a significant increase in the expression of *OsLSi1* and *OsGR*. This increase likely led to greater levels of antioxidant enzymes and a reduction in ROS. These results suggest that SiO_2_NPs suppress the gene expression behaviors of Cd’s absorption and transporter genes in rice during Cd stress. According to previous research, *OsLSi1* enhances antioxidant performance and acts as a favorable transporter gene for silicon in rice [61]. Thus, SiO_2_NPs appear to be an appropriate approach to mitigate Cd stress in single segment substitution line of wild rice.

## 4. Materials and Methods

### 4.1. Experimental Strategy

Hydroponic research was performed at SCAU (South China Agriculture University) Guangzhou China. Single segment substitution line of wild rice (*Oryza glumaepatula*) was used for this experiment, which was prepared by our research group (Figure S1). These lines had good comprehensive agricultural characteristics, strong adaptability, high yield, and high quality. First, we carried out trial-designed research to adjust the doses of silicon dioxide nanoparticles. We used 50 μM, 100 μM, and 150 μM doses of SiO_2_NPs for the preliminary experiment, and selected the 50 μM to proceed with further research. The seeds were sterilized for a half hour using 0.5% NaClO, then rinsed with ddH_2_O and kept in an incubator for 1.5 days at 30 °C. Following that, seeds were stored in petri plates until they were germinated. Upon reaching the 8-day growth stage, wild rice seedlings in good condition were transferred into 1 L plastic pots containing 0.9 L of BNS (basal nutritional solution). The pots were placed in chamber to maintain growth at 16/8 °C (day/night) with a humid environment 65% and a lid with 12 equally sized holes, with one plant per hole. The nutrient solution was aerated constantly and changed every 3rd day. After 12 days of transplantation, 4 treatments were produced by using 50 μM SiO_2_ and 100 μM Cd as CdCl_2_ to the appropriate pots. Culture solutions were updated after every 3 days. Plants were sampled at the seedling stage when they reached 12 days of treatment. The experiments follow the randomized block design with four replicates of each treatment. Then, assessments were made based on the growth of the plant samples and their photosynthetic attributes.

### 4.2. Monitoring of Growth Metrics in Plants

After two weeks of the treatment period, each plant was taken out and divided into shoots and roots, and the length of its roots and shoots was measured. To remove adsorbed ions and potential chemical residue on surfaces, roots were immersed in a solution of 20 mM-EDTA for 25 min. After that, they were rinsed with ddH_2_O, and then the fresh weights of the plants, including roots and shoots, were calculated.

### 4.3. Assessment of Chlorophyll Contents

Fresh plant leaves of wild rice were evaluated for their concentration of carotenoids, chlorophyll a, b, and chlorophyll a + b [62]. Fresh leaves from the treated line were removed and incubated in 95% ethanol under dark conditions for 48 h. The solution absorbance was measured at 470 nm, 649 nm, and 665 nm using a spectrophotometer instrument (UV-1700; Shimadzu, Kyoto, Japan).

### 4.4. Measurement of Oxidative Damage in Rice Plants

MDA and H_2_O_2_ were used to measure oxidative damage in plants. The method used to find the MDA concentration was our previous protocol [62]. 0.5% of TBA (thiobarbituric acid) and 2 mL of TCA (trichloroacetic acid) (*v*/*v*) were used to homogenize the plant samples (0.2 g). Subsequently, the samples were heated for 30 min at 95 degrees Celsius, and then the reaction was stopped by instant cooling using ice. Plant samples were spun for 10 min at 10,000 rpm, and the density at the optical range was measured by spectrophotometer (UV-Vis Spectrophotometer, Hitachi High-Tech Corporation, Kyoto, Japan) at 450 nm, 532 nm, and 600 nm. Using the earlier procedure, the H_2_O_2_ content in rice seedlings was measured [63]. Following a known quantity of fresh plant tissues (0.2 g) being crushed in 0.1% of C_2_HCl_3_O_2_, the mixture was passed through centrifugation for 15 min at 12,000 rpm. After dissolving the upper layer in one milliliter of buffer solution (10 mM KH_2_PO_4_, 500 μL) and 1 mL of 1 molar KI, the absorbance in the spectrophotometer at 390 nm was measured.

### 4.5. Antioxidant Compounds Evaluation

After being digested in a 50 mM Na_3_PO_4_ solution (*v*/*v*) at pH 7.8, fresh samples of plants with a known weight (0.2 g) were centrifuged for a period of 15 min at 13,000 rpm for 4 °C. To assess the defense of CAT, POD, and SOD, their upper surface was taken for measurement. For SOD, POD, and CAT measurements, different kits were used. For SOD analysis, test kit A001-3-2 (WST-1 method) and CAT examination, the A007-1-1 kit (Visible light method) were used to measure the activities of these enzymes [64]. POD functions were calculated by a POD assessment kit (Visible light method, A084-3-1) [55]. Using high performance liquid chromatography, GR level of the plants was determined. All of the processes were carried out according to manual instructions, and the kits were obtained from Nanjing Jiangcheng Bioengineering Institute China.

### 4.6. Characterization of SiO_2_NPs

The 99.9% pure nanoparticles used in this study were purchased from Shanghai (Chaowei Nanotechnology) Co., Ltd., Shanghai, China. The properties of the nanoparticles were identified using XRD, and the morphology and size of the particles were examined using a V 460, FEI (Hillsboro, OR, USA), scanning electron microscope. Prior to applying the coating treatment, conductive tape was placed on the samples, and the SiO_2_ nanoparticles were equally distributed on the conductive tape. Compressed air was then applied to remove any particles that did not adhere to the edge. After that, the samples were placed under a ZEISS EVO MA15 (Jena, Germany) scanning electron microscope for observation. For SEM and XRD, the samples were prepared using the previously established protocol [55]. The particle size distribution of silica samples in aqueous medium was determined by the laser particle size analyzer ZetasizerNanoZS90 (Malvern, UK).

### 4.7. Observation of Cd and SiO_2_NPs Contents in Wild Rice

Following oven drying, the rice root and shoot samples were ground into a fine powder. Then, 0.2 g of each sample was mixed with a 6 mL solution of HNO_3_:HClO_4_, 5:1 *v*/*v*, and the mixture was left overnight to be broken down until only 1 mL of liquid was left. After digestion, the 1 mL sample was diluted with ddH_2_O to a final volume of 50 mL. After the liquid cooled, the concentration of Cd and Si was measured with a spectrophotometer (Model 3200-C, Heinz Walz GmbH, Effecltrich, Germany).

### 4.8. Profound Insights into Transporters and Gene Expression Using qRT-PCR Analysis

Plant samples of roots were systematically gathered once the various treatments were finished. To ensure accuracy in the procedure, total RNA extraction was carried out with RNA extraction test kit and rigorous adherence to the manufacturer’s manual instructions. The reverse transcription of RNA was performed using the Evo M-MLV kit, and the quantitative reaction was performed using the HB210720 (Yisheng RT-PCR kit, Shanghai, China). The next amplification procedure was comprised a two-step procedure with 40 cycles: 5 min of initial denaturation at 95 °C, secondly for 10 s of denaturation at 95 °C, and annealing/extension was carried out at 60 °C for 30 s. The configuration system contained 0.4 μL of primer, 1.0 μL of cDNA template, 8.2 μL of ddH_2_O, and 10 μL of HieffTMq SYBR Green PCR Master Mix and made up the reaction mixture. According to the previously used protocol [65], the Light Cycler 480 fluorescence quantitative PCR machine was used as the instrumental platform (Basel, Switzerland). The gene expression normalization was done using the *Actin* gene as a reference point. Table S1 contains the sequences of the relevant genes *OsABC*, *OsABSG11*, *OsHMA43*, *OsWAK11*, *OsGR, OsLSI,* and *Actin*. Using the 2^−ΔΔCt^ technique, the gene expression patterns were assessed, referring to the previously described protocol [66].

### 4.9. Statistical Analysis

Using the SPSS software (version 23.0), the data were assessed using the LSD test of four replicates. The data were shown using means and SD. The probability status was calculated with values of not more than 0.05.

## 5. Conclusions

The research found that silicon dioxide nanoparticles (SiO_2_NPs) can reduce Cd stress in wild rice. Rice plants treated with 50 µM SiO_2_NPs demonstrated significant improvements in a variety of growth metrics, including the weight, length of roots and shoots, chlorophyll contents and carotenoid levels. This treatment of SiO_2_NPs also increased the activity of antioxidant enzymes while decreasing the hazardous elements in wild rice, such as Cd content, H_2_O_2_, and level of MDA in rice plants. Furthermore, SiO_2_NPs were discovered to reduce Cd toxicity in plants by increasing photosynthetic efficiency, decreasing oxidative damage, and increasing antioxidant enzyme capacity and gene expression level in the Cd stressed environment. Among the several SiO_2_NPs concentrations examined, 50 µM was revealed to be the most effective in lowering Cd stress. Nevertheless, our study suggests that more research is needed to understand the molecular pathways underlying SiO_2_NPs’ impact on improving Cd tolerance in these lines developed from wild rice, and comparative studies should be conducted.

## Data Availability

Data are contained within the article and Supplementary Materials.

## Supplementary Materials

The following supporting information can be downloaded at: https://www.mdpi.com/article/10.3390/plants13121715/s1, Figure S1. Schematic illustration of a line developed from wild rice; Table S1: Primer Gene sequence of metal transporters.
