# Supplementary material for: Silicon Dioxide Nanoparticles-Based Amelioration of Cd Toxicity by Regulating Antioxidant Activity and Photosynthetic Parameters in a Line Developed from Wild Rice
Source: Plants (Basel). 2024 Jun 20;13(12):1715. doi: 10.3390/plants13121715 (PMC11207486; doi:10.3390/plants13121715)
Supplement: Supplementary file 1 [file plants-13-01715-s001.zip › plants-3007806-supplementary.pdf]

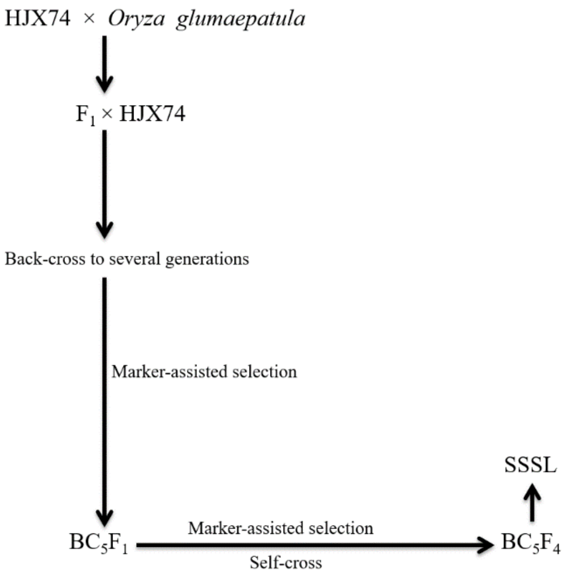

Figure S1. Schematic illustration of a line developed from wild rice.

Table S1. Primer Gene sequence of metal transporters

**Gene**	**Forward primer sequence**	**Reverse Primer sequence**
OsHMA3	ACCTCAGTCAACTCAAGAAA	GTCAGCATAACCGACTTG
OsABC	TCTCCGGGAAGGTTAGGA	CCTCGCTCTCCAATCTCT
OsABC43	TATTTCGAGGCAATTCC	CCACACACTGATTTAGG
OsGR	ACATGTGTGCTTCGTGGGT	GATCAACTATCTTTCCACGG
OsLS1	TCCTCAAGAAGGTCGTGTC	CGTAGATCATCACCGTCAC
OsWAK11	AGGGTGCCAAGATATCAACG	CATACCGAAATGCCTATAAACG
Actin	TGTTATGGTAGGGATGGGTC	TTCTCTCTATTTGCCTTGGG
